# The Endowment Effect and Beliefs About the Market

**DOI:** 10.1037/dec0000143

**Published:** 2020-11-23

**Authors:** Elena Achtypi, Nathaniel J. S. Ashby, Gordon D. A. Brown, Lukasz Walasek, Eldad Yechiam

**Affiliations:** 1 Department of Psychology, University of Warwick; 2 Harrisburg University of Science and Technology; 3 Department of Psychology, University of Warwick; 4 Faculty of Industrial Engineering and Management, Technion–Israel Institute of Technology

**Keywords:** endowment effect, valuation, ownership, market price, good deal

## Abstract

The endowment effect occurs when people assign a higher value to an item they own than to the same item when they do not own it, and this effect is often taken to reflect an ownership-induced change in the intrinsic value people assign to the object. However recent evidence shows that valuations made by buyers and sellers are influenced by market prices provided for the individual products, suggesting a role for beliefs about the markets. Here we elicit individuals’ beliefs about whole distributions of market prices, enabling us to quantify whether or not a given transaction constitutes a “good deal” and to demonstrate how an endowment effect may reflect such considerations. In a meta-analysis and three laboratory experiments, we show for the first time that ownership has no effect on beliefs about either: (a) the quality of the item or (b) the appropriate market price for the item. Instead, we show that sellers demand a price for the item that matches their beliefs about the item’s relative quality and the distribution of market prices in the market. Buyers, in contrast, offer less than what they believe the appropriate market price is. Thus, we argue that the endowment effect may largely reflect “adaptively rational” behavior on the part of both buyers and sellers (given their beliefs about relevant markets) rather than any ownership-induced bias or change in intrinsic preferences.

Owners of a consumer good demand more money in exchange for it than nonowners are prepared to pay to acquire it—this is the endowment effect. The ratio of sellers’ “willingness to accept” (WTA; the amount they would require to part with the object) to buyers’ “willingness to pay” (WTP; the amount they would pay for the object) frequently exceeds two, even when incentives are used to ensure that people do not engage in strategic behavior (Horowitz & McConnell, 2002; Tunçel & Hammitt, 2014). However, the psychological explanation for this ubiquitous effect remains unclear (see Morewedge & Giblin, 2015, for a review). Previous explanations have assumed that the endowment effect reflects bias on the part of sellers, buyers, or both. According to the loss aversion account, sellers who face the prospect of losing their possession demand more in compensation for it than buyers are willing to pay to acquire the same product (Kahneman, Knetsch, & Thaler, 1990) because losses are more psychologically impactful than gains. Several cognitive and affective processes have been identified to underpin this loss aversion. Studies have shown that emotional attachment to, or psychological ownership of, an object drives higher valuations among the sellers (i.e. owners; Morewedge, Shu, Gilbert, & Wilson, 2009; Shu & Peck, 2011; Walasek, Matthews, & Rakow, 2015; Walasek, Rakow, & Matthews, 2017). Other evidence shows that owners and nonowners differ in their cognitive appraisals of an object, with buyers generally focusing more on undesirable features of a product and sellers focusing more on desirable features (Ashby, Dickert, & Glockner, 2012; Ashby, Walasek, & Glöckner, 2015; Ganzach, 1996; Johnson, Häubl, & Keinan, 2007; Nayakankuppam & Mishra, 2005; Pachur & Scheibehenne, 2012).

In this paper, we take a different approach and consider the possibility that the behavior of both buyers and sellers is driven at least in part by their considerations of what constitutes a good deal for them (Isoni, 2011). Rather than assuming that ownership status is a source of an irrational bias, we evaluate whether the amounts demanded by sellers and offered by buyers reflect their personal beliefs about the product’s relative quality together with their beliefs about the product’s appropriate position in the broader distribution of prices in the market. Beliefs about the market may influence buyers and sellers, as sellers will try to avoid selling a product for less than its market worth, while buyers will avoid overpaying for it. We therefore define the “appropriate market price” as the price that the product is expected to cost in the market given its quality.^1^^,^^2^ Our goal is to establish how buying and selling prices in a typical endowment effect experiment relate to people’s beliefs about the broader context of the market for similar goods. In order to quantify pereceived good dealness of consumer goods, we elicit participants’ individual perceptions of the quality of a product that they have the opportunity to buy or sell, and we also elicit their beliefs about the distribution of market prices for that product or product type. This methodology enables us to determine what each person believes the “appropriate” price for a product to be, given their beliefs about market prices for that or similar products. For example, a decision-maker might believe that a coffee mug is of high quality (e.g., at the 80th percentile of the quality distribution) and that the 80th percentile of the distribution of coffee mug prices corresponds to a price of $6.50. We can therefore examine how each person’s WTA or WTP relates to their beliefs about the appropriate price for a product, as well as establishing whether ownership status influences individuals’ perception of quality, their perceptions of market price distribution, or both. We define participants’ perception of “deal goodness” as their belief about difference between the relative ranked position of product’s price within the relevant market price distribution and the relative rank position of its quality to similar products. To illustrate, a coffee mug at the 80th percentile of the quality distribution on offer at the 60th percentile price clearly represents a good deal. A 40th percentile (quality) coffee mug at the 70th percentile price does not. In the present paper, we examine the relationship between this quantification of deal goodness and a person’s WTA or WTP for a given consumer good.

## Literature Review, Meta-Analysis, and Limitations

Our account extends recent suggestions that beliefs about the market may be important in explaining the endowment effect (Brown, 2005; Isoni, 2011). For example, Isoni (2011) suggests that the discrepancy between sellers’ WTAs and buyers’ WTPs reflects not an ownership-induced difference in underlying preferences for a target object but rather aversion to a bad deal. Specifically, buyers are more averse to the possibility of overpaying for an object than they are to potentially missing out on acquiring an object if they fail to offer a high enough price. However, Isoni (2011) does not offer any direct evidence that perception of the target object’s attributes (e.g., its quality and/or the price it would typically be sold for in the marketplace) are uninfluenced by the ownership status, leaving open the possibility of ownership-induced bias in consumers’ perception of such attributes. Weaver and Frederick (2012) offer a related account according to which the endowment effect arises when people’s valuations are lower than the reference price for an object (reasoning that an object’s reference price will often be its market price). When sellers adjust their selling prices to match high reference prices, their valuations no longer reflect only their personal underlying estimates of the value of ownership. Weaver and Frederick (2012) report results consistent with this hypothesis. In a series of studies, they showed their participants retail prices of several products and then asked them to specify how much they would either pay or sell these items for. The results revealed that sellers’ valuations were closer to the retail prices than were the valuations of buyers. For instance, in two conditions, a candy’s price tag was presented to the participants as either $4.00 or $1.49. Mean buying prices for the candy were largely unaffected by the change in the value on a price tag (being $1.54 and $1.20, respectively) while selling prices were highly sensitive to it, being about 80% higher in the high compared to the low price tag condition ($2.88 compared to $1.58). Based on these findings, Weaver and Frederick (2012) concluded that, “Consumers evaluate potential trades with respect to salient reference prices, and selling prices (or trading demands) are elevated because the most common reference prices—market prices—typically exceed valuations” (p. 696).

We surveyed the endowment effect literature for results consistent with those reported by Weaver and Frederick and conducted a meta-analysis. More specifically, we were interested in the differences between market prices and valuations of owners and nonowners of the consumer goods used in individual studies. Our expectation was that valuations of sellers will be typically closer to the market value of an object than valuations of buyers. We searched for studies in which buyers’ and sellers’ valuations were elicited after both groups were shown a product’s market price. In all 13 studies that we found (see Table 1, and online supplemental material for details about literature search), selling prices tended to be closer to the store price than did buying prices. A meta-analysis of these studies indicates that both selling and buying prices fell below the store prices (Cohen’s *d* = 0.42, *z* = 15.88, *p* < .001; Cohen’s *d* = 0.93, *z* = 27.17, *p* < .001, respectively). However, buyers’ downward price deviations from the store price exceeded sellers’ (*Cohen’s d* = −.79, *z* = 13.33, *p* < .001). How can these results be interpreted? On one hand, the fact that sellers’ WTAs are close to the market prices may reflect sellers paying more attention to the broader context of market prices. Consistent with this idea, in endowment effect experiments with monetary gambles (rather than consumer goods) sellers’ valuations tend to be closer than buyers’ valuations to the gambles’ actual expected value (see Yechiam, Ashby, & Pachur, 2017 for a review). Asymmetry of attention toward the context of market prices would also be consistent with studies showing that sellers, but not buyers, are influenced by market price anchors (Simonson & Drolet, 2004). On the other hand, the results in Table 1 may simply reflect the facts that people generally perceive the market price to be too high and that individual valuations reflect endowment-induced differences in preferences (cf. Weaver & Frederick, 2012). In this case, it should not be surprising that WTAs are nearer to the market price than WTPs. Despite these possibilities, it is important to establish how considerations of deal goodness correspond to the decisions made by owners and nonowners.[Table-anchor tbl1]

Both Isoni (2011) and Weaver and Frederick (2012) suggest that the WTA/WTP gap may be influenced by beliefs about and/or considerations of the market prices (see also Brown, 2005). However, in neither study did the authors elicit participants’ individual judgments of the market prices for, or qualities of, objects. This omission is potentially problematic as people may hold different beliefs about the broader distribution of market prices. People may therefore differ in what they believe a product costs and therefore have different opinions on whether a product and its potential price represent a good deal. Crucially, it is possible that ownership status itself can influence participants’ judgments of either the quality of an object or the appropriate price for that object. Such differences could occur if, for example, sellers focused on particularly positive attributes of the consumer good that they own or brought to mind higher prices when considering a reasonable selling price (Ashby et al., 2012, 2015; Carmon & Ariely, 2000; Johnson et al., 2007; Nayakankuppam & Mishra, 2005; Pachur & Scheibehenne, 2012). We offer a novel test of this possibility in Experiment 1, where we elicited estimates of market price distributions for categories of consumer products from individuals after they found out whether they are owners (sellers) or nonowners (buyers) of a consumer product.

We replicated and extended our approach in Experiment 2, in which we reevaluated the endowment effect with respect to the beliefs held by buyers and sellers about the market prices and product’s quality. Specifically, we conducted an incentivized experiment in which we elicited perceptions of quality and market price for a consumer good and then used these quantities to match each individual’s perception of quality onto their beliefs about market price distribution. This process allows us to identify, for each individual, expectations about the appropriate price for a product, given the person’s perception of what the item of a given quality should cost in the broader market. By comparing this quantity to the WTPs and WTAs, we can represent valuations in relations to people’s beliefs about the market. This allows us to determine the extent to which valuations of buyers and sellers are related to their underlying beliefs about market values that constitute a good deal.

In Experiment 3, we address several limitations of Experiment 1 and 2, eliciting market price estimates for a wide range of products and then asking our participants to value these goods as buyers or sellers after a 1 week delay.

To foreshadow our results, Experiment 1 finds that even when sellers demand more for a product than buyers are willing to pay, the two groups share similar beliefs about the broader context of market prices. By recovering the market price position from people’s valuations, we also show that sellers’ WTAs are relatively evenly distributed in the market price distributions. The WTPs of buyers, on the other hand, tend to correspond to the lowest market prices produced by participants. The results of Experiment 3 corroborate these findings as well as the results of our meta-analysis, showing that WTAs are much closer to the elicited market prices than WTPs of buyers. By matching people’s personal beliefs about product quality to their estimates of market price distributions in Experiment 2, we found that ownership status had no effect on people’s beliefs about the quality-appropriate market price (i.e. the *Nth* percentile price for an item judged to be of *Nth* percentile quality) for the relevant item. We also found that the quality-appropriate price for a product was very similar to sellers’ valuations but much higher than buyers’ valuations. Overall, we found that buyers will only pay to acquire a product at a price that represents a good deal and hence will offer considerably less than what they believe the product is worth in the market. Sellers, in contrast, ask for a price close to what they believe the product should cost in the market given their beliefs about its relative quality. These results are consistent with suggestions that buyers and sellers engage in a qualitatively different task—buyers are most concerned about their preferences (typically not wanting an object) and sellers simply attempt to estimate the appropriate selling price given their knowledge of the market (Brown, 2005). We argue that it is possible to explain the behavior of sellers by reference to their individual beliefs about the market and quality. Buyers’ valuations are different, in that they deviate substantially from what the person thinks the product “should” cost in the market given its quality.

## Methodological Statement

In the present article, in addition to the results of a meta-analysis reported earlier, we summarize the results of three laboratory experiments. Throughout the article, we report not just conventional frequentist analyses but also Bayesian analysis, allowing us to quantify evidence for null hypotheses. We performed all Bayesian analyses using JASP (JASP Team, 2020, Version 0.13.1). Our results can be replicated by setting seed to 1 with default priors and the number of samples set (in case of non-parametric tests) to 10,000. Our data and materials are available to other researchers from https://osf.io/jb625/. Institutional ethical approval was obtained prior to data collection.

## Experiment 1

Our initial experiment was modeled on the standard laboratory methods for demonstrating the endowment effect, with some extensions. In addition to collecting WTPs from nonowners and WTAs from owners of an object, we also elicit people’s beliefs about the entire distribution of market prices for a given class of consumer product (here water bottles). This new methodology allows us to assess how WTPs and WTAs compare in terms of their relative position in the person’s internally stored beliefs about the broader market. In particular, in order to assess the notion of deal goodness described in the introduction, we assess how sellers’ and buyers’ valuations rank in the wider distribution of market prices. All exclusion criteria (described below) were decided prior to analyzing the data.

### Method

#### Design

In a between-subjects design, we compared owners’ (sellers) and nonowners’ (buyers) valuations of a university branded water bottle. We also examined their beliefs about the market price distribution of the same object.

#### Participants

We recruited 79 participants using Warwick University’s pool of volunteers (*M*_age_ = 20.70, 59% female). Each individual was promised a flat fee of £3.00 but was told that, depending on their choices, they could earn between £0.00 and £20.00 extra. Each session lasted approximately 20 min.

#### Procedure and materials

Participants were tested in groups of maximum size 10. In any single session, all participants randomly took on the role of either buyers or sellers. Sellers were given a brand-new water bottle with the University of Warwick logo. These bottles were purchased from the University of Warwick bookstore (where they were sold for £6.99). Buyers were told that they would receive an extra £4.00 (£7.00 in total with the flat fee of £3.00) for their participation. At this point, participants were asked to proceed to the next task—production of random patterns on a piece of paper and survey questions about that task, which took on average 10 min to finish.^3^ Once this task was completed, sellers were reminded that they had been given a water bottle and that it was theirs to keep. They were also told that they would have the opportunity to sell the water bottle if they so desired. Buyers were asked to examine the water bottle, which the experimenter had just placed on their desk. They were told that they had the options of either buying the bottle and taking it home with them or keeping all their money.

We used the BDM (Becker, Degroot, & Marschak, 1964) method to elicit valuations. Specifically, participants were informed that at the end of the experiment the computer would generate a random offer/price for the water bottle. On the subsequent screen, sellers were asked the minimum price, in pounds, for which they would be willing to sell their water bottle. Buyers were asked the highest amount of money, in pounds, that they would be willing to pay for the water bottle.

**Figure 1 fig1:**
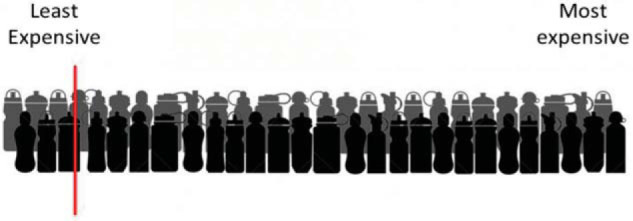
Example question of the elicitation process that was shown to participants.

After specifying their WTA or WTP for the water bottle, we elicited participants’ beliefs about the prices of similar products in the market. Participants were shown an image of two rows of water bottles. They were asked to imagine that these bottles represented all unique bottles in the market and that they were ordered from the cheapest (leftmost bottle in the picture) to the most expensive (rightmost bottle in the picture). Participants were then asked to give their best estimate of the price, in pounds, corresponding to a specific position in the market. We used nine percentiles in total (10, 20, 30, 40, 50, 60, 70, 80, and 90). For each percentile, participants saw a red line indicating a particular position in the market (see Figure 1). They were then asked “The line indicates a price. [percentile]% of all water bottles cost less than the price indicated by the line, and [1-percentile]% cost more. What is the price indicated by the line (in British pounds)?” The nine questions were presented in random order.[Fig-anchor fig1]

The elicitation of the price distribution was incentivized: The three individuals who gave the best (i.e. most accurate) estimates for prices of water bottles were awarded bonus payments of 15.00, 10.00, and 5.00 pounds for the first, second and third places, respectively, after all sessions were concluded. (To enable this, the responses were compared to real price data for water bottles extracted from Amazon.co.uk.—we simply ranked participants with respect to the distance between the mean of the distribution of Amazon prices and the mean of the distribution estimated based on their responses.) After the elicitation of the price distribution, participants were reminded about the BDM procedure and that the computer would now randomly generate an offer/price and compare it with their selling/buying price/offer. The results of the BDM procedure were shown to participants, who were then asked to alert the experimenter. After all transactions were concluded, participants were thanked and debriefed.

### Results

First, we removed data from one participant whose valuation (here WTA) was extremely high (> = four *SDs* from the mean). We also identified and removed responses from two participants who did not provide consistent answers on the distribution elicitation task. Specifically, we calculated Kendall’s τ coefficients to determine whether participants’ responses were monotonically increasing with the percentiles of the distribution. We used a cut-off of 0.7 for this correlation coefficient. The final sample included 36 sellers and 40 buyers.

We found clear evidence of an endowment effect: Sellers demanded a median amount of £4.00 (*Range* = [£0.05, £20.00]) for the bottle whereas buyers were willing to pay only £1.00 (*Range* = [£0.00, 7.50]) to obtain it. The WTA/WTP ratio of the medians (i.e. 4) is comparable to that typically obtained in the endowment effect literature (Horowitz & McConnell, 2002; Tunçel & Hammitt, 2014)^4^. The values of WTA and WTP were found to be significantly different according to a Mann–Whitney *U* test, *W* = 296, *p* < .001, *r* = −0.589, 95% CIs[−0.734, −0.392]. A Bayesian nonparametric test produced decisive evidence for a model where buyers and sellers produce different valuations of the water bottle, BF_10_ = 253.59.

**Figure 2 fig2:**
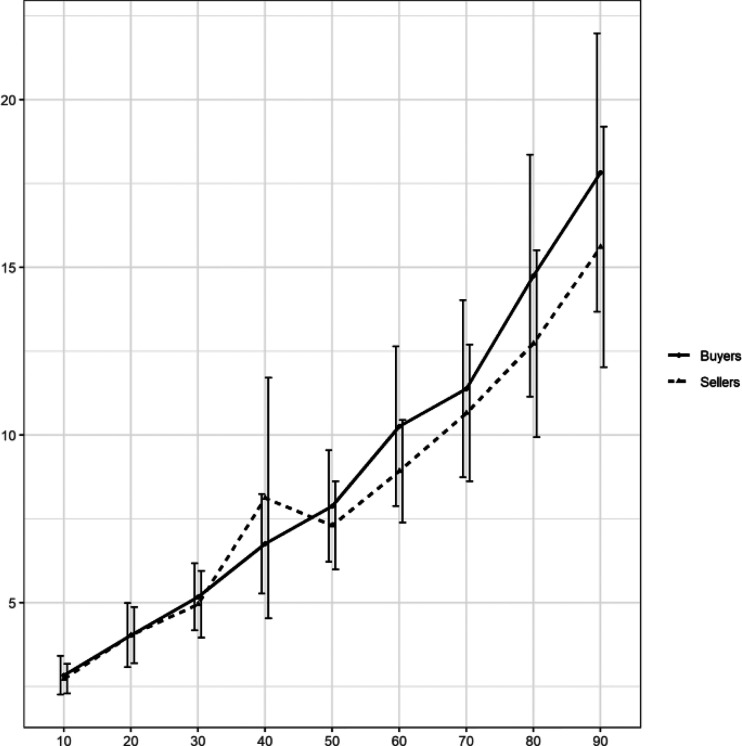
Mean percentile estimates of the market price in Experiment 1. Error bars in the right panel represent ± 2 standard errors of the mean.

Figure 2 summarizes the median and average responses on the distribution elicitation task. It is evident that participants provided similar estimates of the market price distribution of the water bottles. We tested this with a mixed effect analysis of variance (ANOVA with Greenhouse-Geisser correction), including a between-subjects factor for condition (buyer vs. seller), a within-subjects factor for percentile (10 vs. 20, and so on), and their interaction term. We found no main effect of condition with *F*(1, 74) = 0.265, *p* = .608, partial η^2^ = 0.004. As expected there was a main effect of percentile, *F*(2.08, 153.954) = 71.584, *p* < .001, partial η^2^ = 0.492. The interaction term was not significant, *F*(2.08, 153.954) = 1.039, *p* = .358, partial η^2^ = 0.014. For robustness, we also performed a two-sample Kolmogorov–Smirnov test to compare two distributions, *D* = 0.04, *p* = .932.[Fig-anchor fig2]

We conducted a Bayesian version of the mixed effect ANOVA. Here we found decisive evidence in favor of the model that included the main effect of percentile (relative to the null model), with BF_10_ = 2.51 × 10^79^. With respect to the model with the main effect of condition, we found strong support in favor of the null model, with BF_10_ = 0.220. In other words, our data were 1/0.220 = 4.55 more likely to be observed under the null model. Finally, the model with main effects of percentile and condition was supported by data more than the model with the interaction term included, BF_01_ = 1/.022 = 46.20.

What percentiles of the market price distribution do participants’ WTAs and WTPs correspond to? We computed the rank position of each participant’s WTA(P) in their elicited distribution of market prices by fitting a lognormal distribution to each individual’s responses. In the fitting process, we used a least-squares parametric fit to lognormal inverse cumulative density functions (CDFs). We then calculated the rank of each participant’s WTA(P) with that participant’s fitted distribution.

**Figure 3 fig3:**
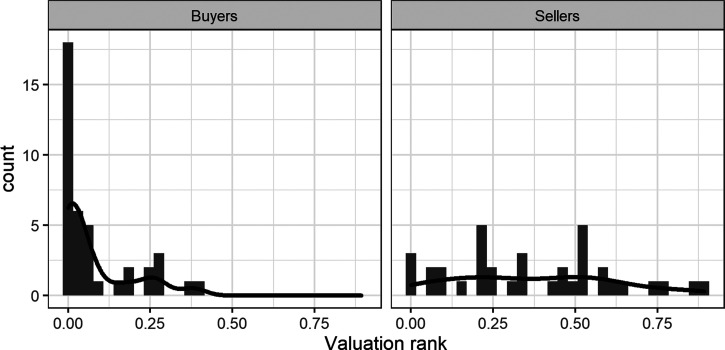
Histograms of WTA(P) ranks within individually fitted market price distributions in Experiment 1.

We found that the average ranked position of sellers’ WTAs was significantly higher (*Median* = 0.339, *Range* = [0; 0.892]) than the rank of buyers’ WTPs (*Median* = 0.029, *Range* = [0; 0.390]), *t* = −6.83, *p* < .001, *d =* −1.569, 95% CIs[−2.082, −1.049]. The Bayes factor of BF_10_ = 4.23 × 10^6^ signifies that the model with different means among sellers and buyers is much more likely to be true than the model in which the two groups have the same means. This is unsurprising given that we observed a large endowment effect in the absence of any differences in elicited market price distributions. All ranks are plotted in Figure 3. First, it is clear that many owners and nonowners provided a valuation that ranked very low in their perceived market price distribution of all water bottles. However, this is particularly apparent among buyers, the majority of whose valuations ranked extremely low in the respective market price distributions. Among sellers, the price demanded for a water bottle corresponded to a wider range of market positions.[Fig-anchor fig3]

### Discussion

In Experiment 1, we replicated the classic endowment effect with an incentive compatible valuation protocol and showed that our distribution elicitation method works well, with only two participants out of 78 failing to provide us with a satisfactory level of monotonicity of market prices. Our results also showed that buyers and sellers do not differ in their perception of market prices. The results therefore provide the first evidence that ownership status does not lead to a distorted perception of market prices, and hence that the endowment effect cannot reflect any such bias. Instead, we found that valuations of buyers and sellers ranked differently in their elicited beliefs about the spread of the market prices for water bottles. Whereas sellers’ valuations reflect a wide range of possible market prices (see Figure 3), the majority of buyers gave valuations that corresponded to the lowest prices in the market.

In Experiment 2, we extend the methodology of Experiment 1 and consider people’s perception of product’s quality. This allows us to quantify the degree to which buyers and sellers differ in their concern with making a good deal.

## Experiment 2

The key objective of Experiment 2 was to understand how people’s WTAs and WTPs relate to their estimates of the quality and market price of the relevant object. Specifically, we wanted to be able to quantify the amount of “deal goodness” required by buyers to purchase an object that they likely have little desire for and the amount of “deal badness” acceptable by sellers when giving up the item. To achieve this, we elicited people’s beliefs (and certainty) about quality and market price, as well as their beliefs about the broader market (as in Experiment 1).

### Method

#### Design

The design of Experiment 2 was identical to that of Experiment 1 with the addition of two questions: one about the person’s perceived quality of the water bottle and one about his or her estimate of the market price for the product. We counterbalanced the order in which these two sections were presented (in a 2 × 2 ANOVA, with condition and ordering as between-subjects variables, ordering was not a significant factor. We therefore do not include ordering in the analyses that follow. See Online Supplemental Table T1, for a summary of the ANOVA).

#### Participants

We recruited 92 participants (*M*_age_ = 21.90, 63% female) from the University of Warwick and tested them in groups of 10 or fewer (not fewer than four per session). Each individual was promised a flat fee of £3.00 and was told that, depending on their choices, they could earn between £0.00 and £20.00 more.

#### Procedures and materials

The procedure was the same as in Experiment 1 but extended to accommodate new measures. Following the market price elicitation task, participants also provided their best estimate of the actual market price for the target water bottle. To measure the uncertainty in these price estimates, participants also specified an upper price (that they were 90% certain the true price was below) and a lower price (that they were 90% certain the true price was above). The inclusion of this measure was motivated by the possibility that buyers and sellers may differ in their uncertainty about the true market price. One possibility is that sellers are less certain in their estimates and hence that their valuation is motivated by the possibility that a product could cost more in the market. We compared the lower and upper bounds of the estimated market prices to test for this possibility.

**Figure 4 fig4:**
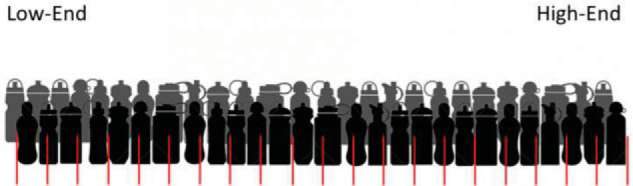
Quality rank question as shown to participants.

Next, we elicited estimates of the quality of the water bottle. Participants were shown a new picture of all water bottles in the market, but now ordered by their quality (see Figure 4). Participants were told that at the high-end, water bottles had the most features and were made of the best materials, and at the low-end, they had the fewest features and were made of the poorest materials. They were then asked to indicate, by clicking on the appropriate region of the scale, where they believed the water bottle that they were given (or offered) ranked in terms of quality.[Fig-anchor fig4]

The rank participants gave was represented by a green rectangle on the graphic. After providing their point estimates participants were asked to give low and high estimates of the water bottle’s quality such that they were 90% sure the water bottle’s quality fell above the low estimate and below the high estimate. These estimates were also made using the graphical interface shown in Figure 4.

### Results

We excluded two participants based on the same criteria as were employed in Experiment 1. None of the participants provided us with extreme WTA/WTP values, but responses of two individuals were removed due to poor consistency in the distribution elicitation task (Kendall τ < 0.7). In total the sample consisted of 47 sellers and 43 buyers.

As in Experiment 1, we found a clear endowment effect. Summary statistics and relevant tests (both frequentist and Bayesian) are reported in Table 2.[Table-anchor tbl2]

The WTA/WTP median ratio of 1.92 was smaller than the ratio of 4 found in Experiment 1, but the difference between the groups was significant, with decisive evidence in support of the model with different valuations of buyers and sellers (BF_10_ > 16).

With respect to the stated market price, we found strong support for there being no difference between the groups. However, sellers provided higher upper bounds for the market price than buyers did. We found no difference for the lower bound of the market price, although the evidence here was not conclusive in the Bayesian analysis.

**Figure 5 fig5:**
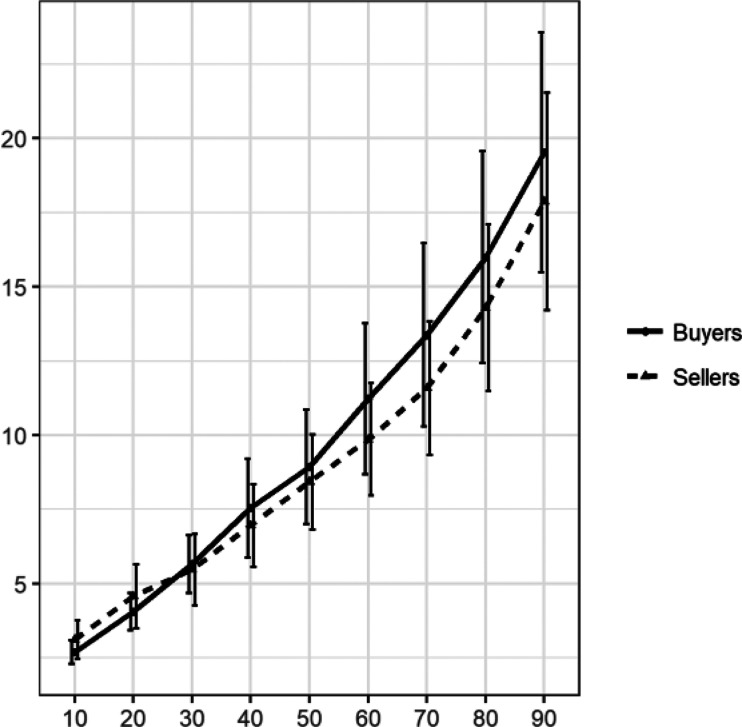
Mean percentile estimates of the market price in Experiment 2. Error bars in the right panel represent ± 2 standard errors of the mean.

Next, we examined responses from the market price elicitation task. Figure 5 shows mean responses for each percentile, separately for buyers and sellers.[Fig-anchor fig5]

Visually, the results closely mimic findings from Experiment 1 (see Figure 2). In a 2 (condition) by 9 (percentile) mixed ANOVA, we find a main effect of percentile, *F*(1.326, 116.677) = 125.205, *p* < .001, partial η^2^ = 0.587, no main effect of condition, *F*(1, 88) = 0.340, *p* = .562, partial η^2^ = 0.004, and no significant interaction, *F*(1.326, 116.677) = 0.941, *p* = .359, partial η^2^ = 0.011. The results of the Kolmogorov–Smirnov test were consistent, with *D* = 0.07, *p* = .276.

The Bayesian version of this test provides us with quantitative support for the null hypothesis. First, we found decisive evidence in favor of the model containing the main effect of percentile, with BF_10_ = 1.045 × 10^126^. However, we found strong support for the null model relative to the model with the main effect of condition: BF_10_ = 0.225. The data were 1/0.225 = 4.44 times more likely under the null model. Lastly, we found strong evidence in favor of the main effect only model over the model with a two-way interaction: BF_01_ = 1/.012 = 83.33.

Taken together, data from Experiment 1 and 2 indicate that the elicited distributions of market prices do not differ between buyers and sellers. The same holds true if people state the market price explicitly. We observe some differences in the stated uncertainty expressed by buyers and sellers, although this effect is small in magnitude.

**Figure 6 fig6:**
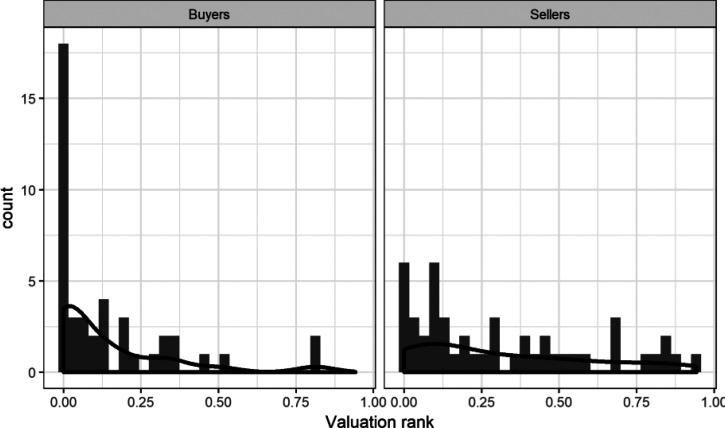
Histograms of WTA(P) ranks within individually fitted market price distributions in Experiment 2.

How did participants’ valuations rank in the elicited market price distributions? For each participant, we fitted a lognormal distribution and calculated the rank position of their WTA/WTP within these distributions of market prices. In Figure 6, ranks of buyers and sellers’ prices are plotted next to each other. The lower section of Table 2 provides descriptive statistics and the results of inferential statistics comparing buyers with sellers.[Fig-anchor fig6]

Consistent with Experiment 1, we find clear evidence of a difference in ranks. Once again, a large proportion of buyers’ valuations had extremely low ranks in the market price distributions, whereas sellers’ prices were more evenly distributed across possible prices. Clearly, acceptable amounts among sellers match onto a range of potential market prices, whereas the maximum that buyers are willing to offer corresponded to very low (often the lowest) market price for similar category of products.

#### Quality rank

Table 2 summarizes the data for the median judged quality rank and for the upper and lower ends of the participant’s 90% confidence cut-offs. Although we found no significant difference between buyers and sellers in judged rank of quality, we found strong evidence that the lower and upper ranks differ between groups: Sellers provided higher cut-offs for the lower and upper end of the quality interval.

#### Good dealness

With the information about people’s valuations, perceived quality ranks, and beliefs about the distribution of market prices, we can examine participants’ implied beliefs about good dealness. Specifically, we can match a person’s quality rank to that person’s market price distribution to obtain the amount of money that a person believes a product should cost in the market.

We computed the quality-matched price in the following way. For each participant, we took their estimate of the ranked quality of the water bottle and found the market price that occupies the corresponding rank position in the distribution that represents that participant’s beliefs about the market. For example, if a person stated that the water bottle was at the 30^th^ percentile for quality, we calculated the 30^th^ percentile price in the distribution of market prices that we elicited from that participant. We refer to this estimate as the quality matched price (QMP). In the same fashion, we obtained the high quality matched price (HQMP) and lower quality matched price (LQMP) that correspond to the lower and upper bounds of the confidence intervals provided by the participant. More formally, if *F*^−1^(*e*; μ, σ) is the fitted lognormal inverse CDF for a participant’s elicited market price distribution, where *e* is the percentile and (μσ) are the parameters for the lognormal distribution, then the quality matched price, QMP, is related to the quality rank QR by: *QMP* = *F*^−1^(*QR*; μ, σ). In the bottom portion of Table 2, we report QMP, HQMP and LQMP, respectively. We found strong support for the suggestion that the QMP and LQMP are no different between buyers and sellers. For HQMP, we also find no difference, but the strength of evidence is less convincing, with Bayes analysis favoring the null model with BF_10_ = 2.00.

It is evident that the estimates of the quality matched prices are very similar to participants’ estimates of the market prices of the product (see Table 2). Buyers and sellers agreed with respect to the market price of the water bottle (median of 5 in both groups), but their beliefs about the appropriate price for the product, given their beliefs about its quality, were very near to these values (with median values of £5.58 for buyers and £5.45 for sellers). There was therefore a high degree of coherence in participants’ estimates: Their direct estimates of the market price for the water bottle correspond to their estimates of the water bottle quality combined with their beliefs about the market price distributions of water bottles’ prices.

**Figure 7 fig7:**
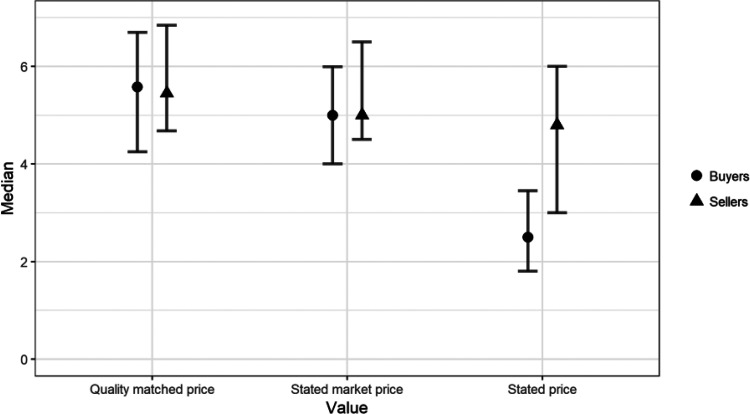
Valuations of buyers and sellers together with elicited market prices and estimated appropriate prices for the water bottle. Error bars represent bootstrapped 95% confidence intervals.

We summarize our results in Figure 7, which shows median stated market prices, quality matched prices, and actual stated valuation (WTA or WTP) of our participants.[Fig-anchor fig7]

We interpret the results in terms of our quantification of deal goodness described earlier. On average, sellers estimated the water bottle to be at the 35^th^ percentile of the quality distribution and were prepared to accept the 21^st^ percentile of the market price distribution for it. Buyers estimated the water bottle to lie at the 30^th^ percentile of the quality distribution yet were prepared to pay merely the 5.5^th^ percentile price. We interpret these results to indicate that buyers are only willing to purchase the good if they can acquire it at a considerable discount/as a good deal.

Further support for the good dealness explanation comes from the correlations between the estimated market prices and valuations provided by our participants. We found that WTPs are positively correlated with stated market prices, *r*(41) = 0.49, *p* < .001, and so are WTAs, *r*(45) = 0.76, *p* < .001.

### Discussion

Experiment 2 replicates findings of Experiment 1, showing that buyers and sellers do not differ with respect to their beliefs about the market price distributions. Once again, ranks of valuations within these distributions differed markedly between the groups, such that buyers were only willing to pay a low market price for the water bottle while sellers’ valuations corresponded to a wider (and higher) range of market prices. Experiment 2 also extends results of Experiment 1 by quantifying good dealness considerations among buyers and sellers. More specifically, we found that buyers and sellers agree on what the water bottle should cost, given its quality and given a broader market context. Yet, buyers were willing to pay much less than these amounts to acquire the water bottle.

## Experiment 3

The results of Experiment 1 and 2 are consistent with the notion that buyers and sellers differ in their consideration of good dealness. However, these studies have a number of limitations, which Experiment 3 seeks to address. First, the duration of ownership differs between the conditions in Experiment 1 and 2. Sellers were exposed to the water bottle for longer than buyers, and this feature of the experimental design can increase the WTA/WTP ratio (Reb & Connolly, 2007; Strahilevitz & Loewenstein, 1998). For this reason, in Experiment 3, the exposure to products was held constant. The second concern is that our experiments were limited to a single consumer good—university-branded water bottles. Although mugs with University logos have been often used in endowment effect studies, it is important to show that our results replicate for other products as well. To address this, Experiment 3 uses a wider range of consumer goods. Third, there is a risk of a carryover effect in our studies, whereby participants provide valuations and market estimates in a single session. In Experiment 3, we separated valuations from estimation by a period of at least two weeks.

Beyond these modifications, Experiment 3 has two more distinct features. First, instead of asking participants to provide a single point estimate of the market price, we elicit a distribution representing participants’ beliefs about the plausible market prices for each product. With this indirect measure of the perceived market price, we then take the midpoint of this distribution as our measure of people’s perceived market value for the consumer good. Our focal hypothesis is that these estimates of the market price will be closer to the valuations of sellers (WTAs) than buyers (WTPs). Second, the results of Experiment 1 and 2 (as well as findings of Weaver & Frederick, 2012) suggest that the BDM procedure may not work as intended, with buyers and sellers considering different things when evaluating consumer goods in a typical experimental setting. It could therefore be that many participants allocated to the condition of buyers are simply uninterested in a particular object and therefore provide valuations very close to £0. To explore this further, we included a binary question asking participants whether they want to engage in a transaction at all before they could provide valuations of the goods (both for buyers and sellers).

### Method

#### Design

Our participants were assigned to the role of a buyer or seller of 10 consumer goods. All individuals were asked to answer questions about market prices for those products and to specify their selling or buying price for each item.

#### Participants

A total of 84 participants took part in the two-session study (*M*_age_ = 24.74; 45% female). Each experimental session lasted approximately 15–30 min. Every individual was given £6.50 in each of the two sessions for their participation (£13.00 total). In addition, to further incentivize their decisions, eight participants were selected at random, and one of their decisions was carried out for real (see below for details).

#### Materials and procedure

**Figure 8 fig8:**
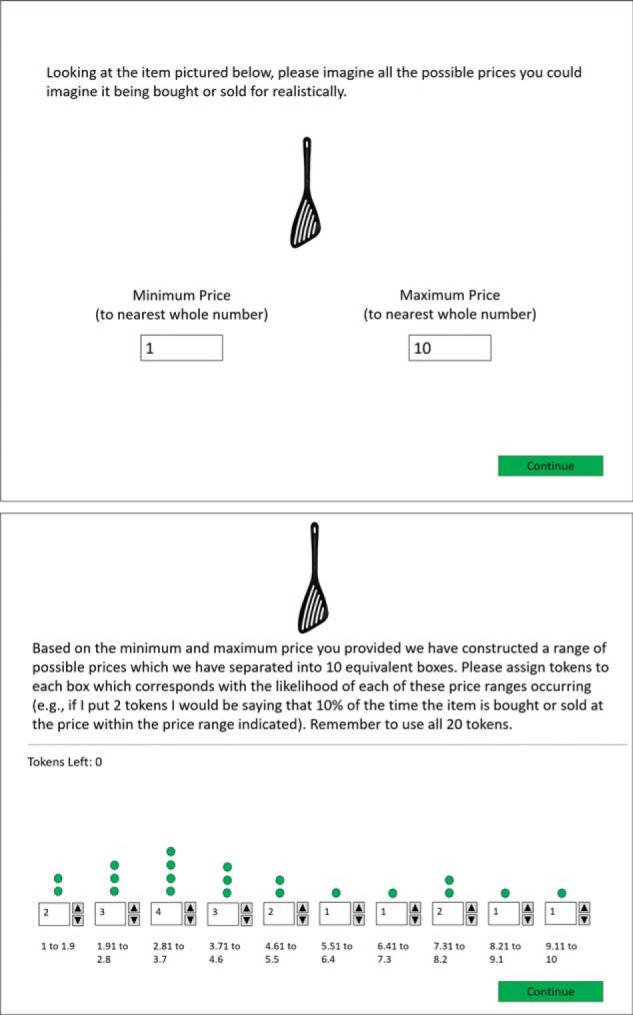
Procedure for estimating distribution of store prices for a single product. Top: first stage, where participants indicated the minimal and maximal store prices. Bottom: second stage, where participants filled in the distribution of equally spaced price intervals.

Products consisted of 10 common consumer goods (e.g., tongs, spatula, plate) found on Amazon.co.uk. All products had a listing price of less than £20.00 and had positive average reviews (i.e. three or more stars on average). The experiment was programmed using Real Studio, 2012 r2.1. The experiment consisted of two sessions. In the first session, which was the same for all participants as they were only subsequently treated as buyers or sellers, participants were presented with an image of each product (in random order) and asked to indicate what they thought the highest and lowest store price for the product was (see top panel of Figure 8). Participants were then asked to provide a distribution of store prices for each product. Specifically, they saw 10 equally spaced bins spanning the range from the low to the high price they had provided earlier. For each bin, participants had to indicate how many out of 100 stores (in five store increments) they expected the product would be sold for in the given price range (see bottom panel of Figure 8). Next, for validation purposes, participants indicated the benefits of the product on a scale ranging from −5 (*totally unbeneficial*) to 5 (*totally beneficial*). After all products were evaluated participants were paid for their time and reminded to attend the second session.[Fig-anchor fig8]

In the second session—which occurred at least 7 (but no more than 20) days after the first session—participants were randomly assigned to the role of either buyer (*N* = 39) or seller (*N* = 45). Buyers were told that there was a 10% chance they would be given £20.00 and that their task was to provide buying prices for 10 different products. When entering each buying price, a checkbox was presented that participants could use to indicate if they did not want to engage in a transaction involving a given product. By checking the box, participants could indicate that they did not want to buy the product at all (equivalent to WTP = 0). If a participant was randomly selected to receive £20.00 at the end of the study, one of the products was selected at random. If they had indicated they did not want to buy the randomly selected product, they were given the £20.00. Otherwise a random amount between 0 and £20.00 was drawn by the program. If the amount drawn was less or equal to the buying price provided, participants received the product and £20.00 minus the amount drawn. If the random amount was greater than their buying price, they were given the £20.00. This method is a variant of the incentive compatible methods of eliciting valuations commonly used in the endowment effect literature (BDM method; Becker et al., 1964).

Sellers were told that there was a 10% chance that one of the products they provided a selling price for would be given to them. As in the buying condition, when entering each selling price, a checkbox was present that allowed participants to indicate if they did not want to sell a given product for any price. If a participant was selected to take part in the transaction at the end of the study, one product was randomly chosen. If they had indicated they did not want to sell the product, it was given to them. Otherwise a random amount between £0.00 and £20.00 was drawn. If the random amount drawn was equal to or larger than their indicated selling price they received the drawn amount; otherwise they received the product.

After providing buying or selling prices for all 10 products, participants were thanked for their time, debriefed, and paid. Buyers and sellers whose decisions were incentivized were informed via e-mail that they could come in and pick up their money and/or product.

### Results

We removed data from one buyer who failed to answer a majority (>80%) of questions. As a result, our experiment contains 83 participants who each provided 10 valuations and 10 estimates of market prices (1,660 responses in total). There were two missing entries in total (0.12%). In seven responses (0.42% of the data), participants’ store prices were more than four standard deviations (SDs) above the mean store price (based on valuations from all participants). These responses were removed from the data. For the analysis of WTP and WTA 3/1,660 responses (0.36% of the data) were similarly excluded due to being four standard deviations above the mean of the elicited price. Thus in total, we had 1,648 valuations and market price estimates.

Overall, products were evaluated as beneficial (*Median* = 1.50; *Range* = [−1.66, 3.26]), with no significant difference in the rated benefit for those participants who were randomly allocated to be buyers (*Median* = 1.36; *Range* = [−1.66, 3.26]) or sellers (*Median* = 1.65; *Range* = [−0.75, 3.05]), *W* = 759, *p* = .383, *r* = −0.112, 95% CIs[−0.348, 0.137]. Moreover, a Bayesian analysis indicated positive evidence for the absence of a difference (BF_10_ = 0.278). Table 3 shows the buying, selling, and estimated store prices for each of the 10 products. Consistent with the endowment effect, the median selling prices (across all products; *Median* = £2.70; *Range* = [£1.22, £8.60]) were higher than the median buying prices (across all products; *Median* = £2.11; *Range* = [£0.60, £5.10]). We tested this using a 2 (condition: buyers vs. sellers) by 10 (product type) mixed effect analysis of variance (ANOVA). In the analysis, we used log transformed valuations due to non-normality of people’s stated buying and selling prices. The results revealed a main effect of ownership status, *F*(1, 79) = 4.385, *p* = .039, partial η^2^ = 0.053, and a significant main effect of product type, *F*(6.95, 549.07) = 14.00, *p* < .001, partial η^2^ = 0.151. There was no significant interaction between these variables, indicating that the size of the endowment effect did not vary across the product types, *F*(6.95, 549.07) = 1.256, *p* = .270, partial η^2^ = 0.016. Taken together, we found that sellers always demanded more for the products than buyers were willing to pay, but the difference was rather small.[Table-anchor tbl3]

The results of the Bayesian ANOVA are consistent with these results. There was strong support for the model with the effect of product type, with BF_10_ = 1.029 × 10^18^. There was strong support for the model including ownership status and product type main effects, with BF_10_ = 1.633 × 10^18^. The model with main effects only was 33.44 times more likely than the model with main effects and the interaction.

We next examined the relation between valuations and estimated store prices. Table 3 shows the median valuation and median store prices for each product. Across all products, medians for WTPs and WTAs were always lower than the median estimates of the market price. We compared differences between valuations (WTPs and WTAs) and market prices using one sample *t* tests (*H*_0_ being that the difference is 0). Relevant Bayes factors are reported in the Table *3*, showing substantial evidence, for all products but one, that the differences between WTPs and market prices are larger than 0 (here also negative). In contrast, for eight products we found substantial evidence in the favor of the null hypothesis, supporting the prediction that there is no difference between valuation (WTA) and estimates of the market price. Together, these results are largely consistent with the previous finding showing that selling prices are close to the products’ market prices.

We further examined whether the price of a given product (relative to other products) is associated more strongly with the median store price for sellers than for buyers. We computed correlations to test whether sellers or buyers who indicated high median store price also indicated higher WTA/WTP across different products. The median estimated store prices were correlated with valuations of sellers, *r*(444) = 0.63, *p* < .001 and, to a lesser extent, with valuations of buyers, *r*(370) = 0.57, *p* < .001.

Finally, we analyzed the number of products that participants indicated that they wanted to buy or sell at the price they provided. Sellers chose to sell a median of 4 (*Range* = [0, 9]) out of the 10 products, while buyers chose to buy a median of 1 (*Range* = [0, 10]) products; a significant difference, *W* = 1312, *p* < .001, *r* = 0.535, 95% CIs[0.332, 0.690], BF_10_ = 99.003. Therefore, buyers not only departed from the market price of the products but also displayed lower willingness to consider an exchange.

### Discussion

Across a wide range of products, we find that the valuations of sellers are nearer to their perceived market worth than the valuations of buyers are. This is in line with the results of our meta-analysis. Notably, however, we demonstrate this finding remains after controlling for the amount of exposure to the product experienced by buyers and sellers and using an indirect method of eliciting the point estimate of the product’s market price. In addition, we find that buyers are more likely not to want to engage in the transaction than sellers. In other words, buyers indicate that they are not interested in the transaction at all more often than sellers do.

## General Discussion

We explored the hypothesis that valuations of buyers and sellers may reflect their differing beliefs about the broader market of prices and products (Brown, 2005; Isoni, 2011; Weaver & Frederick, 2012). Specifically, we developed a novel quantification of deal goodness in terms of the rank-based difference between the “appropriate” price (generated by the quality matched process) and the WTA and WTP monetary valuations. In terms of this good-dealness consideration, the endowment effect emerges because, given they will typically lack any strong desire to possess the object, buyers are only willing to purchase a product if they get a very good deal (relatively low market price given products’ quality). Sellers valuations, on the other hand, should correspond closely to the expected market price for a given good. Indeed, we show that sellers are willing to accept prices that correspond to their beliefs about what the given product should cost in the broader market. Buyers do not differ significantly from sellers in their beliefs about the market but are willing to pay substantially less than they believe the product costs in the market.

In Experiment 1, using a distribution elicitation task, we set out to determine whether beliefs about the distributions of market prices for a given class of consumer products (here water bottles) differ as a function of ownership status. We found no evidence of such a bias (see also Experiment 3 in Walasek, Yu, & Lagnado, 2018). In addition, we demonstrated that while both buyers and sellers value the object at less than its market price, buyers have a strong tendency to provide WTP amounts that correspond to the lowest end of the market price distribution. In Experiment 2, we replicated these findings and additionally found that owners and nonowners do not differ in their estimates of the product’s quality (in terms of how its quality ranks among other similar products). Moreover, owners and nonowners produced similar estimates of the product’s actual market price. Using a wide range of consumer products, valuations of sellers in Experiment 3 were closer to the estimated market price of each good than valuations of buyers were.

Our results are comparable to the findings reported in studies of the endowment effect for risky and ambiguous gambles. Sellers, not buyers, tend to set the minimum selling price to be close to the actual objective worth of a risky asset (Yechiam, Abofol, & Pachur, 2017; Yechiam et al., 2017). Although in the present study we cannot make any statement about the ideal price of a consumer good for each person, our findings show that sellers’ valuations align with their perception of what the item should be worth as a product in the marketplace. Our results therefore extend previous efforts beyond the context of gambles.

Our study builds on and extends recent accounts suggesting that the endowment effect is at least in part driven by the considerations of what constitutes a good deal. This account differs from traditional explanations of the endowment effect in several key respects. Most importantly, unlike many accounts based on concepts such as loss aversion, our account does not assume ownership-induced changes in people’s valuations of the object if such valuation is defined in terms of underlying preferences (rather than, e.g., the profit that could possibly be made by selling it). In this respect, our account is similar to that of Isoni (2011). However, unlike Isoni, we do not need to assume “bad deal aversion” in that we do not require any asymmetry in hedonic impact of under- and overpaying. Of course, we do not discount the possibility that ownership status can influence people’s valuations via mechanisms such as asymmetric attention (Ashby, Walasek, & Glöckner, 2015; Carmon & Ariely, 2000) or psychological ownership (Walasek et al., 2015; Walasek et al., 2017).

It is important to note that our results do not provide direct causal evidence for the relation between perceptions of good dealness and valuations of owners and nonowners. Instead, our account is mostly descriptive—we illustrate how valuations of buyers and sellers map onto participants’ beliefs about the product and the broader context of the consumer market. By doing so, we can show patterns of valuations that fit well with recent theoretical and experimental developments in which the behavior of buyers and sellers is largely dictated by their consideration of how to secure (avoid) a good (bad) deal. Past work and our own results thus align with a simple pragmatic explanation of the endowment effect. When participants come to the lab and are offered a chance to purchase some consumer good, most people do not want it, even at a substantial discount (relative to its potential market value). Sellers on the other hand, value the product appropriately given on their knowledge of the market. If this account correctly captures people’s reasoning, there is no need to invoke any psychological biases, such as loss aversion, to explain the endowment effect. We do not provide direct evidence against loss aversion explanation of the endowment effect but rather offer an alternative explanation of the valuation gap. The conclusion of most researchers (i.e. that the endowment effect reflects loss aversion) is based on the assumption that participants’ valuations reveal their true underlying preferences, which are in turn assumed to be uncontaminated by strategic considerations or beliefs about the market. This assumption stands in contrast with the finding that even in an incentivized experiment, many buyers and sellers admit that their valuations were motivated by “seeking a good deal” or a consideration of a “reasonable or compromise price” and “selling cheaply to make sale likely” (for sellers, Brown, 2005). Further research is necessary to show how buyers and sellers might be differently influenced by their beliefs about the broader market. One potential extension of the present study would be to manipulate beliefs about the market. Using products that are less known among the participants, we would expect that valuations of sellers would be much more influenced than those of buyers by such a manipulation. The evidence presented by Weaver and Frederick (2012) is consistent with this prediction. Although our results do not disprove a role of loss aversion in the endowment effect, our alternative account suggests that the assumption of loss aversion is not necessary. We therefore suggest that explanations of the endowment effect in terms of loss aversion be discounted until and unless specific evidence for loss aversion is forthcoming. We argue that our account is parsimonious because it explains both the endowment effect and sellers’ greater sensitivity to observed market prices in terms of the difference between buyers’ and sellers’ beliefs about relevant markets without requiring the additional assumption of loss aversion (as postulated for instance by Weaver & Frederick, 2012).

In Experiments 2 and 3, we found that buyers and sellers did not differ in terms of how they rated the products on quality and benefit, respectively. These findings appear difficult to reconcile with the idea that endowment effect emerges, at least in part, due to the sense of emotional attachment that develops among owners (Shu & Peck, 2011; Walasek et al., 2015). Indeed, participants who own an object have been shown in other studies to rate an object more favorably than nonowners—a phenomenon known as the mere ownership effect (Beggan, 1992). One plausible explanation for our results is that our design did not provide owners with enough opportunity (or reason) to develop any meaningful sense of psychological ownership. Even in the case of Experiment 2, where owners had more contact with the product than nonowners, such a short duration of ownership could simply be insufficient to generate any special bond between an individual and a consumer good.

The idea that perception of good dealness is an important influence on stated buying and selling prices has wider implications concerning the use of incentive compatible procedure like the BDM (Becker et al., 1964) to elicit true valuations. If the amount that people are willing to sell or buy an item for reflects market considerations relating to appropriate prices for an item of that quality, rather than or as well as an individual’s desire to possess the object, the valuations obtained using BDM-like procedures cannot be interpreted as unbiased measures of underlying preferences. At the very least, our results suggest that sellers and buyers engage in the valuation task differently, with sellers intuitively considering broader context of the market in making their decisions.

## Supplementary Material

10.1037/dec0000143.supp

## Figures and Tables

**Table 1 tbl1:** Studies Comparing Selling Prices (Willingness-to-Accept; WTA) and Buying Prices (Willingness-to-Pay; WTP) of Consumer Products Where Participants Were Provided With the Store Price Before Making Their Pricing Decision

Study	Product	N_WTA/WTP_	WTA	WTP	Store price
Kahneman, Knetsch, & Thaler, 1990, Exp 1	Pen	22/22	2.06	0.75	$3.98
Kahneman et al., 1990, Exp. 7	Mug	39/39	7.00	2.00	$6.00
Morrison, 2000	Mug	10/10	2.20	0.99	£1.90
Morrison, 2000	Chocolate bar	10/10	0.31	0.29	£0.33
Arlen, Spitzer, & Talley, 2002	Mug	18/17	4.71	3.14	$5.95
Roth, 2006	Metro ticket	29/28	1.79 (0.15)	1.33 (0.29)	€2.20
Weber et al., 2007^w^	Song tracks (self-chosen)	16/16	1.21 (0.89)	0.51 (0.32)	€1.29
Knutson et al., 2008^w^	Computer gadgets	24/24	45.87 (13.83)	22.58 (10.37)	$70.57
Weaver & Frederick, 2012, Study 1+	Candy	55/70	2.23 (1.31)	1.37 (0.93)	$2.75
Weaver & Frederick, 2012, Study 2+	Mechanical pencil	77/78	1.17 (1.18)	0.80 (0.71)	$1.54
Weaver & Frederick, 2012^w^, Study 4+	Chocolate bar	40/40	5.23 (1.88)	3.17 (1.48)	$7.50
Abofol, 2016	Grocery products	31/34	8.06 (2.48)	5.95 (3.15)	₪7.93
Gal and Rucker (2017)	Watch, notebook, mug, phone	246/262	166.82 (58.63)	116.50 (54.77)	$177
*Note.* The columns denote the product type, the mean WTA and WTP (in the same currency as the store price), and the store price. Between-subject standard deviations appear in parenthesis. N_WTA/WTP_ is the ratio of the number of valuations made by owners to the number made by non-owners.					
^w^ = Within-subject design (same individuals performing buying and selling). ^+^ = Averaged across conditions with the same product but with varying price tags.					

**Table 2 tbl2:** Summary Statistics and Pairwise Comparisons for Stated and Inferred Valuations of the Water Bottles in Experiment 2

Outcome variable	Buyers:	Sellers	Mann-Whitney U test	Bayes factor (BF_10_)
	Median [Range]	Median [Range]	(*W, p*)	
Stated values				
Stated price	£2.50 [£0.00; £8.50]	£4.80 [£0.00; £15.00]	*W* = 616.5, *p* = .001	16.149
Market price	£5.00 [£1.00; £12.95]	£5.00 [£1.50; £15.00]	*W* = 958.5, *p* = .676	0.254
Lower market price	£3.00 [£0.50; £12.00]	£3.00 [£0.00; £90.00]	*W* = 829.5, *p* = .142	0.457
Upper market price	£8.00 [£1.00; £20.00]	£9.00 [£3.50; £50.00]	*W* = 741.5, *p* = .029	3.338
Quality rank	0.3 [0.1; 0.7]	0.35 [0.1; 0.8]	*W* = 822.5, *p* = .128	0.718
Lower quality rank	0.15 [0.05; 0.35]	0.20 [0.05; 0.80]	*W* = 771.5, *p* = .052	1.440
Upper quality rank	0.45 [0.15; 1]	0.75 [0.20; 1]	*W* = 641.5, *p* = .003	10.137
Inferred values				
Valuation rank	0.055 [0; 0.814]	0.212 [0; 0.942]	*W* = 581.0, *p* < .001	28.527
QMP	£5.58 [£1.57; £21.57]	£5.45 [£1.00; £14.93]	*W* = 950.0, *p* = .628	0.246
LQMP	£3.93 [£1.12; £18.93]	£4.25 [£0.73; £13.97]	*W* = 918.0, *p* = .459	0.263
HQMP	£8.58 [£2.09; £31.52]	£8.96 [£1.37; £57.92]	*W* = 876.0, *p* = .281	0.397
*Note*. QMP = quality matched price; LQMP = lower quality matched price; HQMP = high quality matched price.				

**Table 3 tbl3:** Results of Experiment 3: Median Selling Prices (Willingness-to-Accept; WTA), Buying Prices (Willingness-to-Pay; WTP), Median Estimated Store Prices Based on the Distributions Reported by the Participants, Median Differences Between Valuations and Store Prices

Product	Median WTA	Median Market	Median of differences	BF_10_	BF_01_	Median WTP	Median Market	Median difference	BF_10_
1. plate	2.50	3.23	−0.33	0.305	3.276	2.00	2.91	−0.68**	0.615
2. spoon	2.00	2.60	−0.27	0.174	5.733	2.00	2.51	−1.01**	31.179
3. wineglass	4.00	3.70	−0.61	0.193	5.183	3.00	3.81	−1.09***	155.519
4. spatula	2.50	2.51	−0.02	0.163	6.116	2.00	2.74	−0.76***	91.113
5. tongs	3.00	3.11	−0.59*	2.488	0.402	2.50	3.71	−1.39***	339.513
6. spatula (2)	2.00	2.36	−0.20	0.214	4.668	1.90	2.44	−0.79***	874.438
7. bowl	3.00	3.01	−0.33	0.632	1.581	2.05	3.41	−1.31**	3.474
8. dish scrubber	1.50	2.01	−0.51*	0.944	1.059	1.50	1.80	−0.60**	9.784
9. coffee cup	3.00	2.91	−0.01	0.166	6.026	2.00	3.06	−0.69**	39.735
10. rolling pin	3.00	3.26	−0.51*	2.973	0.336	2.40	3.39	−0.84***	258.807
*Note*. In order to simplify the interpretation of the Bayes factors, we included a transformation such that BF_01_ = 1/BF_10_ when testing the difference between sellers’ valuations and stated market prices.									
For the one sample Wilcoxon signed-rank tests: * *p* < .05. ** *p* < .01. *** *p* < .001.									

## Footnotes

1 Throughout the paper we use the term “appropriate” when referring to the market price that one would expect to pay for an item. It is not a reference to people’s internal preferences, as a person may not want to pay as much as the product costs in the shop.

2 We acknowledge that there may be many attributes and reasons underlying participants’ valuations. In our studies, we elicit ratings of benefit and quality as these are general enough to encompass a variety of such considerations.

3 Note that we did not ask sellers to do anything with the water bottle—it was left next to them during the filler task. We come back to this design feature in Experiment 3.

4 Note that many researchers tend to report means and standard deviations, and the mean WTA/WTP ratios are typically closer to 2. However, stated valuations are typically positively skewed, and medians are therefore better summaries of the central tendencies.
