# Supplementary material for: The Endowment Effect and Beliefs About the Market
Source: Decision (Wash D C ). 2020 Nov 23;8(1):16–35. doi: 10.1037/dec0000143 (PMC7983076; doi:10.1037/dec0000143)
Supplement: Supplementary file 1 [file DEC-2019-0049_Supplemental_Materials.docx]

**Appendix I. Meta-analysis of past literature**

We reviewed the literature to evaluate whether sellers are more sensitive than buyers to market prices when those prices are presented just before the pricing decision. To find the relevant articles indicated in Table 1, we employed a Google Scholar search, using the keywords “WTA” (Willingness to Accept), “WTP” (Willingness to Pay), and either “price tag”, “store price”, or “at the store” (last search May 2017). Additionally, we included all relevant articles reported in a recent meta-analysis on buyer-seller differences in pricing of consumer goods (Tunçel and Hammitt 2014). We also submitted announcements to the listservs of the Society for Judgment and Decision Making and the Economic Science Association. No restrictions were applied in terms of language.

Our search revealed 259 results for “WTA” + “WTP” + “price tag” and an additional 23 results for + “store price” and 26 results for + “at the store”. Additionally, 6 articles were sent to us following submitted announcements. The abstracts of these articles were scanned and 14 were found to be relevant in that they compared buying and selling prices. Among them, 9 papers indicated the store price prior to the pricing decisions. In Table 1 we report the 13 independent studies in these papers. Means and standard deviations of different products within a study were pooled. To examine the respective differences, we used the Mantel-Haenszel method (Mantel and Haenszel 1959; Greenland and Robins 1985), which is a corrected inverse variance procedure.

As indicated in Table 1, selling prices were closer to store prices than buying prices in all 13 studies that were identified, with buying prices showing a downward departure from store prices.

Greenland, Sander, and James M. Robins. 1985. “Estimation of a Common Effect Parameter from Sparse Follow-up Data.” *Biometrics* 41 (1): 55. https://doi.org/10.2307/2530643.

Mantel, Nathan, and William Haenszel. 1959. “Statistical Aspects of the Analysis of Data from Retrospective Studies of Disease.” *JNCI: Journal of the National Cancer Institute* 22 (4): 719–48. https://doi.org/10.1093/jnci/22.4.719.

Tunçel, Tuba, and James K. Hammitt. 2014. “A New Meta-Analysis on the WTP/WTA Disparity.” *Journal of Environmental Economics and Management* 68 (1): 175–87. https://doi.org/10.1016/j.jeem.2014.06.001.

**Appendix II. Experiment 2 ANOVA with order as a factor.**

**Table T1. ANOVA results for Experiment 2 including the effect of presentation order.**

| Within subjects effects |  |  |  |
| --- | --- | --- | --- |
|  | *df* | *F* | *p* |
| Percentile | 1.339 | 124.325 | < .001 |
| Percentile * Ownership | 1.339 | 1.077 | 0.321 |
| Percentile * Order | 1.339 | 3.663 | 0.046 |
| Percentile * Order * Condition | 1.339 | 0.064 | 0.868 |
| Residual | 115.12 |  |  |
|  |  |  |  |
| Between subjects effects |  |  |  |
|  |  |  |  |
| Ownership | 1 | 0.481 | 0.490 |
| Ordering | 1 | 3.739 | 0.056 |
| Ownership * Ordering | 1 | 0.159 | 0.691 |
| Residual | 86 |  |  |
